# Supplementary material for: Severe infections in peritoneal dialysis and home hemodialysis patients: An inception cohort study
Source: PLoS One. 2023 Jun 14;18(6):e0286579. doi: 10.1371/journal.pone.0286579 (PMC10266644; doi:10.1371/journal.pone.0286579)
Supplement: S1 Table — (DOCX) [file pone.0286579.s001.docx]

| S1 Table. Characteristics of home dialysis patients | | | | | | | |
| --- | --- | --- | --- | --- | --- | --- | --- |
|  | Home dialysis modality | | | | | Missing values (%) | Used as imputation predictor |
|  | CAPD | APD | | Home HD | |  |  |
| Number of patients | 162 | 229 | | 145 | | 0.0 | No |
| Male (%) | 70 | 66 | | 68 | | 0.0 | Yes |
| Number of deaths in 5 years^†^ | 66 | 26 | | 12 | | 0.0 | No |
| Primary renal disease (%) ^a, b, c^ |  |  | |  | | 0.0 | Yes |
| Glomerulonephritis | 17 | 22 | | 24 | |  |  |
| Cystic kidney disease | 6 | 15 | | 31 | |  |  |
| Type 1 diabetes | 19 | 21 | | 17 | |  |  |
| Type 2 diabetes | 20 | 9 | | 5 | |  |  |
| Interstitial nephritis | 4 | 4 | | 1 | |  |  |
| Hypertension | 5 | 4 | | 2 | |  |  |
| Unknown | 20 | 13 | | 10 | |  |  |
| Others | 11 | 12 | | 9 | |  |  |
| Comorbid conditions (%) | | | | | | | |
| Acute myocardial infarction | 9 | | 4 | | 5 | ^#^ | Yes |
| Coronary intervention (CABG/PCI) | 11 | | 7 | | 4 | ^#^ | No |
| Angina pectoris / CAD | 7 | | 8 | | 0.7 | ^#^ | No |
| CAD diagnosed with stress test,  perfusion or angiography | 4 | | 3 | | 3 | ^#^ | No |
| Stroke | 9 | | 4 | | 1 | ^#^ | Yes |
| Intracranial bleeding | 2 | | 2 | | 2 | ^#^ | No |
| Transient ischemic attack | 4 | | 1 | | 1 | ^#^ | No |
| Amputation | 2 | | 2 | | 1 | ^#^ | No |
| PVD with intravascular treatment | 4 | | 2 | | 3 | ^#^ | No |
| PVD diagnosed clinically and/or  by noninvasive tests | 5 | | 1 | | 2 | ^#^ | No |
| Congestive heart failure | 9 | | 5 | | 3 | ^#^ | No |
| Malignancy ^a, b, c^ | 11 | | 5 | | 10 | ^#^ | Yes |
| Type 1 diabetes | 21 | | 21 | | 17 | ^#^ | No |
| Type 2 diabetes ^b^ | 21 | | 9 | | 10 | ^#^ | No |
| Liver disease | 1 | | 4 | | 2 | ^#^ | No |
| COPD / Asthma | 9 | | 8 | | 10 | ^#^ | No |
| Gastrointestinal bleeding | 0 | | 0 | | 0.7 | ^#^ | No |
| Dementia | 0.6 | | 0 | | 0 | ^#^ | No |
| Connective tissue disease | 0 | | 0.9 | | 0 | ^#^ | No |
| Vasculitis | 1 | | 2 | | 1 | ^#^ | No |
| Depression | 8 | | 12 | | 9 | ^#^ | No |
| Hypertension ^b^ | 82 | | 84 | | 89 | ^#^ | Yes |
| HIV | 1 | | 0 | | 0 | ^#^ | No |
| AIDS | 0 | | 0 | | 0 | ^#^ | No |
| Dyslipidemia | 41 | | 41 | | 41 | ^#^ | No |
| Parkinson Disease | 1 | | 0 | | 0 | ^#^ | No |
| Sarcoidosis | 0.6 | | 0 | | 0.7 | ^#^ | No |
| Obesity ^a, b, c^ | 22 | | 17 | | 34 | ^#^ | No |
| Other rheumatologic diseases | 4 | | 1 | | 0 | ^#^ | No |
| Other immunological diseases | 0.6 | | 0.9 | | 1 | ^#^ | No |
| Other psychological/neurological/  developmental diseases | 6 | | 5 | | 3 | ^#^ | No |
| Atrial fibrillation ^b^ | 15 | | 5 | | 5 | ^#^ | No |
| Hearing problems | 6 | | 3 | | 1 | ^#^ | No |
| Visual problems ^a, b, c^ | 29 | | 26 | | 14 | ^#^ | No |
| Patients with >= 3 comorbidities (%) | 67 | | 54 | | 52 | ^#^ | No |
| Current smoker | 15 | | 11 | | 18 | ^#^ | No |
| Support in daily activities ^b^ | 30 | | 9 | | 8 | ^#^ | No |
| Living alone | 22 | | 17 | | 19 | ^#^ | Yes |
| Compliance problem ^a, b, c^  No | 14 | | 15 | | 10 | ^#^ | No |
| Dialysis assistance (%) ^†^ |  | |  | |  |  | No |
| by professional | 4.9 | | 1.7 | | 0.7 | ^#^ |  |
| by family member | 18.5 | | 4.4 | | 4.1 | ^#^ |  |
| KTx-listed (%) ^†^ | 39 | | 75 | | 74 | 0.0 | No |
| KTx (%) ^†^ | 25 | | 58 | | 66 | 0.0 | No |
|  | | | | | | | |
| Continuous variables (median) | | | | | | | |
| Age (years) ^a, b, c^ | 65 | | 50 | | 50 | 0.0 | Yes |
| Echocardiography | | | | | | | |
| Ejection fraction | 60 | | 62 | | 65 | 10 | Yes |
| LVEDD (mm) | 49 | | 50 | | 52 | 10 | Yes |
| LVPW (mm) | 11 | | 11 | | 11 | 21 | Yes |
| Septum (mm) | 12 | | 12 | | 12 | 18 | Yes |
| Electrocardiogram | | | | | | | |
| Heart rate (bpm) | 67 | | 67 | | 68 | 5.4 | Yes |
| PR-interval (ms) | 176 | | 162 | | 161 | 12 | Yes |
| QRS-duration (ms) | 94 | | 96 | | 94 | 5.4 | Yes |
| QT-interval (ms) ^a, c^ | 416 | | 412 | | 404 | 5.4 | Yes |
| Corrected QT-interval (ms) | 435 | | 435 | | 430 | 5.4 | Yes |
| P-Axis (degree) | 51 | | 53 | | 51 | 13 | Yes |
| R-Axis (degree) | 17 | | 22 | | 19 | 5.4 | Yes |
| T-Axis (degree) | 54 | | 49 | | 48 | 5.6 | Yes |
| Laboratory findings | | | | | | | |
| P-Creatinine (µmol/l) ^b^ | 558 | | 583 | | 621 | 0.37 | Yes |
| P-Urea (mmol/l) | 26 | | 27 | | 28 | 0.56 | Yes |
| P-Albumin (g/l) ^a, b, c^ | 36 | | 36 | | 36 | 1.3 | Yes |
| P-ionized calcium (mmol/l) ^b, c^ | 1.2 | | 1.2 | | 1.2 | 0.56 | Yes |
| P-Phosphate (mmol/l) | 1.7 | | 1.7 | | 1.8 | 0.37 | Yes |
| Hemoglobin (g/l) ^b^ | 114 | | 111 | | 109 | 1.3 | Yes |
| P-C-reactive protein (mg/l) ^a, b, c^ | 5 | | 3 | | 4 | 5.0 | Yes |
| Total P-Cholesterol (mmol/l) ^a^ | 4.0 | | 4.2 | | 3.9 | 9.5 | Yes |
| High density lipoprotein (mmol/l) | 1.3 | | 1.3 | | 1.2 | 9.5 | Yes |
| P-Triglycerides (mmol/l) ^b^ | 1.6 | | 1.4 | | 1.4 | 9.3 | Yes |
| Systolic BP (mmHg) ^a, c^ | 148 | | 144 | | 148 | 4.7 | Yes |
| Diastolic BP (mmHg) ^a, c^ | 85 | | 89 | | 84 | 4.7 | Yes |
| Height (cm) ^b^ | 170 | | 173 | | 174 | 3.4 | Yes |
| Weight (kg) | 75 | | 77 | | 80 | 2.1 | Yes |
| Body mass index (kg/m^2^) | 26 | | 25 | | 26 | 4.7 | Yes |
| Abbreviations: HD, hemodialysis; PD, peritoneal dialysis; APD, automated PD; CAPD, continuous ambulatory PD; (%), (percentage); CABG, coronary artery bypass grafting; PCI, percutaneous coronary intervention; CAD, coronary artery disease; PVD, peripheral vascular disease; COPD, chronic obstructive pulmonary disease; BP, blood pressure; LVEDD, left ventricular end diastolic diameter; LVPW, left ventricular posterior wall; KTx, kidney transplantation. ^#^No missing values because data were collected as found or not found in patient files. *During the follow-up period. ^†^Not included in propensity scores. Characteristics used in calculation of propensity scores, a: APD vs home HD, b: CAPD vs home HD, c: PD vs home HD. | | | | | | | |
