# Supplementary material for: Severe infections in peritoneal dialysis and home hemodialysis patients: An inception cohort study
Source: PLoS One. 2023 Jun 14;18(6):e0286579. doi: 10.1371/journal.pone.0286579 (PMC10266644; doi:10.1371/journal.pone.0286579)
Supplement: Attachment — Submitted filename: Response to Reviewers.docx [file pone.0286579.s003.docx]

Rebuttal letter

We thank the academic editor and all reviewers for your great effort, which has really helped us to improve this article.

Comments from Academic Editor

I really appreciate your hard work and tremendous improvement. However, there are some concerns that need to be addressed, explicit.

1. The authors should manage confounders with double adjustment for propensity score (focusing pre Rx indication/contraindication)+prognostic score (focusing post Rx prognosis).

2. Please provide your justification with clear reasoning/supporting evidence for comments from the Reviewer 2 and 3.

Authors’ response:

Thank you very much for your helpful comments and for inviting us to submit a revised version.

1. In order to confound a relation between exposure (here dialysis modality) and outcome (infection event) a variable has to be associated with both the exposure and the outcome. If it is not associated with one of these two, confounding is not possible. The propensity score was constructed from variables (all measured before start of dialysis) that were independently associated with the exposure and it included variables related to indication and contraindications for the dialysis modalities. Such variables were primary renal disease (especially polycystic degeneration less common among PD patients), obesity (favored choice of home HD), visual problems (favored choice of PD), compliance problems (favored choice of PD), high age (favored choice of CAPD), need for support in daily activities (favored CAPD). All these variables were included in the propensity score. Other indication variables were evaluated as well (see list of all variables in Supplementary S1 Table), but not included as they were not significant. Thus, the currently used propensity score already focuses on pre-Rx indication/contraindication variables. In addition, the propensity scores include some other variables that may be connected to prognosis (that is risk of infection episode). These are for example: malignancy, hypertension, atrial fibrillation, albumin, CRP, and hemoglobin.

According to your suggestion, we have now added to the adjustment prognostic variables that were not included in the propensity score. To identify such prognostic variables, we included all variables in Supplementary S1 Table that had not been included in the propensity score in a stepwise forward Cox regression analysis. In this way, we were able to identify the prognostic variables that had not been included in the propensity score. We then performed a sensitivity analysis with adjustment for both the propensity score and the prognostic variables. The methodology of this analysis has been described on lines 154–159. We added the result of this analysis on lines 190 and 193–196. This analysis did not change the main result of our study.

2. Please see our answers to reviewers 2 and 3.

Reviewer #2

Reviewer’s comment: The authors have fully and satisfactorily addressed my main concerns. I still have a feeling that the study compares therapies which are very difficult to match, but I appreciate that a maximum effort has been made in the statistical analysis to correct for biases, and one cannot expect to go further under a retrospective design. The explanations given in the Discussion concerning the limitations of the study seem sufficient to me.

Authors’ response: Thank you for your comment. You are right. Observational studies always have a risk of confounding and bias. As it has not been possible to randomize patients into dialysis modalities, evidence has to be sought from observational studies. At least three issues support that there is a causal relation between dialysis modality and infection burden if looking at the Bradford Hill criteria for causality:

1) Strength in effect size with a hazard ratio of >2
2) Temporality: before start of dialysis there was no difference in rate of infection episodes between patients who later started PD or home HD
3) Plausibility: It is plausible that PD patients have larger infection burden than home HD patients because of their peritonitis risk. Even if randomizing patients to PD or home HD it is likely that the patients randomized to PD would have higher peritonitis risk than those randomized to home HD.

Reviewer #3

Reviewer’s comments: This is an observational cohort which aimed to compare infection complications between home hemodialysis versus home peritoneal dialysis.

1. The present home dialysis population are divergent. Even the best statistical methods cannot adjust the dichotomous distribution of the patient populations.

2. The use of CRP is not validated and is contrary to ISPD peritonitis guidelines.

3. The conclusion based on the present data is unjustified.

Authors’ response:

Thank you for your comments.

1) The reviewer is right. Observational studies always have a risk of confounding and bias. Unfortunately, it has not been possible to randomize patients into dialysis modalities. Thus, evidence has to be sought from observational studies. A number of issues support that there is a causal relation between dialysis modality and infection burden in our study according to the Bradford Hill criteria for causality. Please see our answer to Reviewer 2.
2) Thank you for this question. We have mentioned in the article that CRP has not been validated. The CRP definition was especially useful in the current study in order to identify all possibly severe infections, because in Finland, CRP is always measured when a severe infection is suspected. For all patients with an elevated CRP, we further ensured from the patient files that CRP was elevated due to an infection. Although not shown among home dialysis patients, earlier studies have indicated that high CRP correlates with severity of infections, and infection mortality in hemodialysis patients. Our study gives evidence that CRP level is connected to infection severity also among home dialysis patients as CRP level associated with longer hospitalization and higher need for intensive care, and in case of peritonitis, with removal of PD catheter.

The aim of our study was not to study peritonitis specifically, but all types of severe infection episodes occurring among home dialysis patients. The peritonitis rate (and the rate of other infections) would have been higher if not restricting to infections with a CRP higher than 100 mg/l. It is correct that CRP is not mentioned in the ISPD guidelines. We used CRP to identify patients with various types of severe infections in this retrospective cohort study. However, we do not suggest that CRP should be used for clinical identification of peritonitis among PD patients.

3) Our conclusion is: “To conclude, CAPD and APD patients in our study had higher risk of severe infections than home HD patients. This was explained by PD-associated peritonitis. Thus, our results emphasize the importance of peritonitis prevention measures for PD patients.” We refer to our explanations to the editor and both reviewers. We think this conclusion is justified based on the data presented.
